# Gut-brain pathogenesis of post-acute COVID-19 neurocognitive symptoms

**DOI:** 10.3389/fnins.2023.1232480

**Published:** 2023-09-28

**Authors:** Allison M. Plummer, Yvette L. Matos, Henry C. Lin, Sephira G. Ryman, Aleksandr Birg, Davin K. Quinn, Alisha N. Parada, Andrei A. Vakhtin

**Affiliations:** ^1^School of Public Health and Tropical Medicine, Tulane University, New Orleans, LA, United States; ^2^The Mind Research Network/Lovelace Biomedical and Environmental Research Institute, Albuquerque, NM, United States; ^3^Division of Gastroenterology and Hepatology, University of New Mexico, Albuquerque, NM, United States; ^4^Section of Gastroenterology, New Mexico Veterans Affairs Health Care System, Albuquerque, NM, United States; ^5^Nene and Jamie Koch Comprehensive Movement Disorder Center, Department of Neurology, University of New Mexico, Albuquerque, NM, United States; ^6^Department of Psychiatry and Behavioral Sciences, University of New Mexico School of Medicine, Albuquerque, NM, United States; ^7^Division of Internal Medicine, University of New Mexico School of Medicine, Albuquerque, NM, United States

**Keywords:** COVID-19, post-acute COVID-19 syndrome (PACS), post-acute sequelae of COVID-19 (PASC), gut-brain axis, neuroinflammation, endotoxicity

## Abstract

Approximately one third of non-hospitalized coronavirus disease of 2019 (COVID-19) patients report chronic symptoms after recovering from the acute stage of severe acute respiratory syndrome coronavirus 2 (SARS-CoV-2) infection. Some of the most persistent and common complaints of this post-acute COVID-19 syndrome (PACS) are cognitive in nature, described subjectively as “brain fog” and also objectively measured as deficits in executive function, working memory, attention, and processing speed. The mechanisms of these chronic cognitive sequelae are currently not understood. SARS-CoV-2 inflicts damage to cerebral blood vessels and the intestinal wall by binding to angiotensin-converting enzyme 2 (ACE2) receptors and also by evoking production of high levels of systemic cytokines, compromising the brain’s neurovascular unit, degrading the intestinal barrier, and potentially increasing the permeability of both to harmful substances. Such substances are hypothesized to be produced in the gut by pathogenic microbiota that, given the profound effects COVID-19 has on the gastrointestinal system, may fourish as a result of intestinal post-COVID-19 dysbiosis. COVID-19 may therefore create a scenario in which neurotoxic and neuroinflammatory substances readily proliferate from the gut lumen and encounter a weakened neurovascular unit, gaining access to the brain and subsequently producing cognitive deficits. Here, we review this proposed PACS pathogenesis along the gut-brain axis, while also identifying specific methodologies that are currently available to experimentally measure each individual component of the model.

## 1. Introduction

The coronavirus disease of 2019 (COVID-19) is caused by the severe acute respiratory syndrome coronavirus 2 (SARS-CoV-2), and has the hallmark clinical presentation of pulmonary complications that range from mild hypoxia to pneumonia to acute respiratory distress (Johnson et al., 2020). Given the potentially life-threatening severities of COVID-19, most of the research immediately following the 2019 COVID-19 outbreak was focused on the acute disease stage. However, reports of lasting effects began to emerge shortly after the beginning of the pandemic, leading the Centers for Disease Control (CDC) to define “long COVID-19” as symptoms lasting beyond 3 months (Raman et al., 2022). Such effects were quickly found to not necessarily limit themselves to the lungs. Many of the persistent symptoms involve the central nervous and the gastrointestinal systems, including fatigue, brain fog, irritable bowel syndrome, and dyspepsia (Peiris et al., 2021; Choudhury et al., 2022). Here we discuss a multi-system constellation of residual SARS-CoV-2 effects that can remain after the acute stage and may lead to the emergence and maintenance of chronic COVID-19 symptoms. Specifically, we focus on the lasting neurocognitive sequelae that often follow COVID-19, and provide an overview of their proposed pathogenesis along the gut-brain axis.

Subsequent to the acute stage of COVID-19, up to 30% of patients report long-term symptoms, despite the initial infection being resolved and the virus no longer being detectable in the body (Davis et al., 2021). As such, while the epidemiological waves of SARS-CoV-2 have come and gone, the number of individuals suffering from its chronic sequelae has been increasing cumulatively during and after the pandemic, with the CDC estimating that approximately 1 in 13 adults in the US have long COVID-19 at the time of this writing. The pathogenesis of these residual effects of COVID-19 remains unclear, and few tools are currently available to address this looming global health crisis. As such, the described gut-brain model may motivate novel applications for existing gastrointestinal therapies in order to indirectly influence the central nervous system, potentially alleviating the neurocognitive sequelae of COVID-19. We note that the CDC recently approved an ICD-10 code for Post-Acute Sequelae of COVID-19 (PASC); however, other definitions such as Post-Acute COVID-19 Syndrome (PACS) and Post COVID-19 Conditions (PCC) are also common in clinical and scientific communications. With long COVID nomenclature remaining somewhat ambiguous, the authors of this manuscript refer to the condition as PACS.

## 2. Gut-brain axis model of PACS

Described initially as general symptoms and conditions that continue or develop after SARS-CoV-2 infection, the definitive clinical presentation of long COVID-19 remains elusive. Cognitive issues are frequently reported, including memory loss and brain fog (Han et al., 2022), with patients over the age of 65 reporting more severe acute- and chronic-stage symptomology (Mueller et al., 2020; Han et al., 2022). Long COVID also appears to involve developing new-onset conditions such as diabetes, thrombotic, and cerebrovascular disease (Xie et al., 2022). As discussed below in more detail, there is compelling evidence that the SARS-CoV-2 virus compromises intestinal epithelium and cerebrovascular endothelium (Buzhdygan et al., 2020; Cardinale et al., 2020) – both directly and indirectly – lending support to the PACS pathogenesis model based on dysfunctional gut-brain interactions due to damage to the neurovascular unit and intestinal barrier. Specifically, angiotensin-converting enzyme 2 (ACE2) receptors that SARS-CoV-2 relies on for cellular entry are widely expressed by cerebrovascular endothelial and smooth muscle cells (Hamming et al., 2004; Lu et al., 2020; Zhou et al., 2020), and have been implicated in both direct viral damage to the neurovascular unit (Buzhdygan et al., 2020) and indirect damage *via* virus-induced cytokine storms (Elyaspour et al., 2021; Nicosia et al., 2021). The intestinal wall is also rich with ACE2 receptors, making it vulnerable to viral infiltration that can damage the intestinal barrier and increase its systemic permeability (Cardinale et al., 2020). Further, common COVID-19 gastrointestinal disturbances often produce dysbiosis – a shift toward pathological, pro-inflammatory, and potentially neurotoxic gut microbiota (Kopel et al., 2020; Venzon et al., 2021; Yeoh et al., 2021). Collectively, COVID-19 may thus create a scenario in which neurotoxic and neuroinflammatory substances systemically permeate from the gut lumen through the damaged intestinal barrier and produce chronic cognitive symptoms of PACS by accessing the brain *via* weakened cerebral vasculature – damaged both directly by the virus as well as indirectly via dysregulated cytokine activity.

## 3. Neuronal and cerebrovascular components of PACS

### 3.1. Regarding direct SARS-CoV-2 infiltration of the brain

We note that while most investigations to-date have failed to detect widespread SARS-CoV-2 distributions in neuronal or glial cells, some evidence exists of viral entry into the central nervous system *via* the olfactory bulb – a path that may be related to anosmia that is common in COVID-19 (Boldrini et al., 2021; Meinhardt et al., 2021). Specifically, several autopsy studies have provided evidence for SARS-CoV-2 tropism from the olfactory bulb into the brainstem – a brain region that has a relatively high expression of ACE2 receptors (Lukiw et al., 2022). Such evidence includes brainstem neurodegeneration and presence of viral RNA in the brainstem, with higher concentrations specifically in the medullar subregion (Matschke et al., 2020; Solomon et al., 2020; von Weyhern et al., 2020). However, brainstem neuropathology has also been described in deceased COVID-19 patients’ brains with no detectable viral RNA to accompany such findings, giving credence to hypotheses of indirect neurological damage (Al-Dalahmah et al., 2020; Deigendesch et al., 2020; Fabbri et al., 2020). Direct viral pathogenesis for chronic long COVID-19 is particularly unlikely since the acute disease that precedes PACS is often mild in its severity, motivating our indirect model along the gut-brain axis. Such indirect pathogenic effects can occur due to several COVID-19 comorbidities that have well-established neurological sequelae, and include neuroinflammation, metabolic abnormalities, and cerebrovascular dysfunction (Benghanem et al., 2020; Guedj et al., 2021; Solomon, 2021; Yong, 2021; Philippens et al., 2022).

The aforementioned brainstem findings in long COVID-19 are consistent with its cognitive symptomology that largely revolves around fatigue and brain fog, reflecting prior reports of brainstem abnormalities in chronic fatigue syndrome (CFS) (Zhang et al., 2020a,b; Zhang Y. et al., 2021; Hugon et al., 2022; Thapaliya et al., 2023). Other neuroimaging findings are consistent with this clinical presentation as well, with diffusion weighted imaging (DWI) based microstructural changes reported in the thalamus – another region extensively implicated in CFS (Heine et al., 2023). Using DWI, post-COVID-19 changes have also been reported in multiple major white matter bundles that connect distant cortical parts of the brain, including the corona radiata, corticospinal tract, corpus callosum, arcuate fasciculus, cingulate, fornix, inferior fronto-occipital fasciculus, inferior longitudinal fasciculus, superior longitudinal fasciculus, and uncinate fasciculus (Bispo et al., 2022). In addition to microstructural changes, MRI-based volumetric and density effects of COVID-19 have also been reported across multiple brain regions; however, effect directions have been inconsistent (Harapan and Yoo, 2021; Kamasak et al., 2023). Nevertheless, such neuroimaging-based findings have been consistently reported following mild COVID-19 cases, and the extent of such brain changes appear to reflect the severities of acute-stage disease (Qin et al., 2021; Abbasi, 2022; Taruffi et al., 2023). However, with direct SARS-CoV-2 infection of the brain being an unlikely culprit in PACS, the process by which the virus produces its neurological sequelae remains unclear, motivating the indirect gut-brain axis hypothesis described here.

### 3.2. Cerebrovascular damage and inflammation

Despite its apparent multi-faceted effects on the central nervous system, it remains unknown how COVID-19 produces its neurocognitive deficits. Early post-mortem investigations of fatal COVID-19 cases suggested that direct infections may indeed occur, yet these cases followed severe and rapid disease progressions, and were accompanied by classic symptoms of brain infections such as encephalitis and cerebral hemorrhage (Siow et al., 2021; Song et al., 2021). In contrast, we argue that damage to the cerebrovascular endothelium plays a major role in underpinning the various sequelae of COVID-19 in the brain. Early histopathological studies of severe COVID-19 showed endothelial injury that co-localized with cerebrovascular expression of angiotensin-converting enzyme 2 (ACE2) receptors as well as the resulting viral interactions with the tissue (Hamming et al., 2004; Zhang L. et al., 2021). SARS-CoV-2 thus appears to directly bind to the endothelium, possibly compromising the blood brain barrier (BBB) and exposing the brain to harmful substances that may be present systemically, activating immune resident cells and leading to neuroinflammation (Almutairi et al., 2021; Theoharides, 2022). While such systemic compounds are described in further detail below, we note that the viral spike protein itself has been speculated to permeate the damaged BBB and to be involved in microglial activation, with subsequent chronic neuroinflammation likely leading to neuronal damage and contributing to PACS neurocognitive effects such as fatigue and brain fog (Boldrini et al., 2021; Theoharides, 2022).

Existing work also highlights the role of perivascular inflammation in endothelial dysfunction after COVID-19 (Huertas et al., 2020). Proinflammatory cytokines such as TNF-alpha, IL-6, and IL-4 are abundantly released in the context of systemic inflammation associated with acute-stage SARS-CoV-2 infection (Gubernatorova et al., 2020), and often remain elevated chronically as well (Gubernatorova et al., 2020; Queiroz et al., 2022; Schultheiß et al., 2022). Monocyte-derived macrophages (MDMs) help maintain such inflammatory profiles long-term, leaving pro-inflammatory imprints in monocytes that produce long-lasting, aberrant immune responses in patients who recovered from COVID-19, with such MDM responses common even in patients who experience mild COVD-19 (Bohnacker et al., 2022). Specifically, SARS-COV-2 spike protein stimulation produces profound cytokine release in macrophages derived from COVID-19 patients, but not those derived from controls who were never infected (Theobald et al., 2021). Leukotrienes (LTs) produced by white blood cells have also been reported to induce and elevate levels of chemokine ligand 2 and fatty acid synthesis in monocytes and leukocytes, suggesting that enhanced LT synthesis may drive exaggerated pro-inflammatory chemokine responses in post COVID-19 MDMs (Pacheco et al., 2007; Merad and Martin, 2020; Ranjbar et al., 2022). Indeed, this long-term presence of proinflammatory compounds in itself has been shown to inflict damage to cerebrovascular endothelium following COVID-19 as well as other infections, exposing the brain to systemic harmful substances and virions *via* the compromised blood–brain barrier (BBB) (Daniels et al., 2014; Bermejo-Martin et al., 2020; Elyaspour et al., 2021; Nicosia et al., 2021).

Microglial release of cytokines further compounds the inflammatory effects on cerebrovascular endothelium described above, also downregulating astrocytic and pre-synaptic expression of glutamate transporter-1. This in turn leads to disruption of glutamatergic pathways that can potentially induce neuronal excitotoxicity, degeneration, and death (Crunfli et al., 2020; Alenazy et al., 2022). The role of such glutamatergic dysfunction has recently been implicated in PACS neurocognitive symptomology specifically within the dorsolateral prefrontal cortex (Yesilkaya et al., 2021) – a brain region that is part of the executive function network and is involved in complex integration of multimodal information (Vakhtin et al., 2014). Further, the resulting endothelial dysfunction in the brain would be expected to disrupt cerebrovascular autoregulation of blood flow on a broader level, producing lingering neurocognitive effects due to neuronal malnourishment (Nicosia et al., 2021). Crucially, this notion is supported by the increased rates of other, more severe types cerebrovascular pathology associated with COVID-19 such as stroke and microhemorrhage (Fitsiori et al., 2020; Fridman et al., 2020; Sashindranath and Nandurkar, 2021). Collectively, endothelial dysfunction in PACS likely maintains its neurological sequelae *via* at least the following three ways: 1) *exposure* of the brain *via* compromised BBB to harmful substances that 2) *accumulate* due to inadequate cerebrovascular mechanical action necessary for glymphatic removal of byproducts, reflecting blood dysregulation that 3) *malnourishes* brain tissue and leads to neuronal dysfunction.

### 3.3. Neuroimaging of cerebral vasculature and Neuroinflammation

Non-invasive assessment of the neurovascular unit remains elusive. While using gadolinium enhanced imaging to assess blood–brain barrier integrity for research purposes has become controversial due to its toxicity (Rogosnitzky and Branch, 2016), novel techniques such as diffusion-prepared arterial spin labeled perfusion are emerging (Shao et al., 2019). A promising technique for assessing the integrity of the neurovascular unit indirectly is MRI-based cerebrovascular reactivity (CVR) imaging, which measures the hemodynamic response to hypercapnia – increased partial pressure of blood CO_2_ – that induces vasodilation-related changes in cerebral blood flow (CBF) (Lu et al., 2014). Breathing a weak gas mixture of CO_2_, a rapid and potent vasodilator, during neuroimaging allows for measurement of a functional vascular response, which stands in contrast to static CBF measures obtained using arterial spin labeling and transcranial Doppler ultrasound. Like conventional functional MRI, CVR also offers voxel-wise quantification of reactivity. In light of these advantages, the technique has seen increased utility in pathologies related to brain vasculature, such as stroke (Fierstra et al., 2018), small vessel disease (Smith and Beaudin, 2018), traumatic brain injury (Dodd et al., 2020), and age-related cognitive impairments (Sur et al., 2020). Given the profound multi-organ vascular effects of COVID-19, CVR is an ideal tool for examining cerebrovascular function in PACS patients. Quantifying systemic Glial Fibrillary Acidic Protein (GFAP) may complement neuroimaging methods such as CVR in assessing the neurovascular unit, specifically with regard to astrocytic roles in BBB integrity. Astrocytes contribute necessary, non-redundant roles in neurovascular unit maintenance by regulating intercellular endothelial associations in BBB tight junction formations and by linking cerebrovascular endothelial blood flux with neurons (Alvarez et al., 2013; Heithoff et al., 2021). Astrocytic activation responds to multiple pathologies involving BBB disruption, and upregulation of astrocytic cytoskeletal protein GFAP is the hallmark of such reactive astrogliosis (Pekny and Nilsson, 2005).

Despite the great utility of positron emission tomography (PET) for measuring neuroinflammation *in-vivo* for clinical purposes, concerns about its invasive nature as well as high costs often preclude the use of PET in research settings. Non-invasive assessment of neuroinflammation thus remains difficult. Recent MRI-based methods, however, show promise for quantifying regional microglial activation – a part of the innate immune response. Specifically, diffusion weighted magnetic resonance spectroscopy (DW-MRS) can be used to quantify the diffusivity of individual metabolites in the brain (Genovese et al., 2021). The sequence detects microglial activation using apparent diffusion coefficient of cholinergic compounds (ADC_Cho_), which reside in microglial cells in concentrations 3x higher than other brain cells (Urenjak et al., 1993). As microglia undergo morphological changes during activation, changing from a highly branched state to a more spherical one, cholinergic metabolites are able to diffuse more freely within these cells, resulting in higher ADC_Cho_ values detected in microglia-rich brain regions such as the thalamus (Schubert et al., 2021). Importantly, injections of lipopolysaccharide – a potent inflammatory endotoxin – have been demonstrated to induce elevated ADC_Cho_ in the thalamus (de Marco et al., 2022). DW-MRS may thus be an attractive tool for measuring neuroinflammation in the context of this PACS model along the gut-brain axis.

## 4. Gastrointestinal components of PACS

### 4.1. Dysbiosis

The gastrointestinal tract is the largest immune organ within the body, with microbiota regulating host immunity in conjunction with the intestinal mucosal layer (Toor et al., 2019). Although the main target of COVID-19 is the respiratory tract, several lines of evidence point toward substantial involvement of the gastrointestinal tract (Lamers et al., 2020; Meringer and Mehandru, 2022). Gut microbial dysbiosis – a decrease in the diversity of gut flora that allows pathogenic strains to become overrepresented – likely underpins the production of harmful byproducts in the intestine, which can subsequently accumulate within the gut lumen and proliferate systemically *via* a weakened intestinal barrier (Toor et al., 2019). Given that severe gastrointestinal disturbances are common in the acute stage of COVID-19, there is strong evidence for the resulting gut microbiota dysbiosis that can persist long-term (Villapol, 2020). Crucially, symptoms such as diarrhea also commonly occur in patients who have mild cases of infection and do not require hospitalization (Han et al., 2020). Indeed, microbiome examinations in PACS patients 6 months post-infection revealed decreases in diversity, suggesting that COVID-19-induced dysbiosis may persist chronically and maintain inflammation long-term (Chen et al., 2022; Giannos and Prokopidis, 2022).

Compelling evidence for the role of gut dysbiosis in PACS has been emerging *via* both observational and interventional studies, partially motivated by its relatively established contributions to chronic fatigue syndrome – a condition that shares several of its hallmark symptoms with PACS (Guo et al., 2023; Xiong et al., 2023). Indeed, deficits of beneficial intestinal microbial strains and increased pathogen concentrations have been reported in both severe and mild COVID-19 patient groups, as well as across acute and chronic disease stages. Blooms of opportunistic and harmful bacteria, including those with antibiotic-resistance, have been reported in hospitalized COVID-19 patients, increasing their susceptibility to multi-drug resistant infections and mortality from septic shock to rates as high as 57% (Grasselli et al., 2021; Nori et al., 2021). In addition, COVID-19 has been associated with decreased microbial production of short-chain fatty acids such as butyrate – an important energy source for colonocyte metabolism and maintenance of colonic mucosal health. Deficits in butyrate-producing bacteria, such as *Faecalibacterium prausnitzii,* have been shown to reflect acute-stage COVID-19 severities and be associated with systemic proinflammatory cytokine concentrations (Reinold et al., 2021; Yeoh et al., 2021). This microbiome under-representation of *F. prausnitzii* was found to persist chronically in PACS patients as well, with PACS symptom severities being inversely associated with gut levels of *F. prausnitzii* and *Bifidobacterium pseudocatenulatum* – another butyrate-producing bacterial strain (Liu et al., 2022).

### 4.2. Detection of intestinal dysbiosis

While gut dysbiosis can be assessed using stool samples, this can present challenges in research settings. Lactulose breath testing offers non-invasive assessment of gut microbiota that does not require collection of stool samples, and uses hydrogen and methane concentrations in the breath to detect imbalances in gut microbial flora (Bond et al., 1971). Production of H_2_ *via* gut bacterial fermentation is abundant – about 13 L/day (Hartmann et al., 2000; Wolf et al., 2016). While 60–70% is excreted, the rest is used by other gut bacteria (Christl et al., 1992), producing CH_4_ by reducing CO_2_, CH_4_O, and C_2_H_3_O_2_ (Triantafyllou et al., 2014). In humans, H_2_ and CH_4_ are entirely microbially-derived, have no biological roles (Rezaie et al., 2017), and readily diffuse into blood *via* the intestinal wall, which allows them to be measured non-invasively in exhaled breath (Bond et al., 1971; Levitt, 1971). Indeed, H_2_ and CH_4_ production in response to lactulose – an indigestible starch that gets fermented in the colon in a dose-dependent manner (Bond and Levitt, 1972; Corazza et al., 1993) – can detect a form of dysbiosis called small intestinal bacterial overgrowth (SIBO) (Rezaie et al., 2017). These two gasses are routinely measured in the clinical setting to estimate gut dysbiosis using a Lactulose Breath Test. A Lactulose Breath Test (LBT) positive for H_2_ (>20 ppm over baseline) (Rezaie et al., 2017) indicates dysbiosis associated with diarrhea (Chen et al., 2018), while a positive CH_4_ result (>10 ppm over baseline) (Rezaie et al., 2017) reflects methanogen overgrowth related to constipation (Eckburg et al., 2005; Hwang et al., 2010; Pimentel et al., 2020). Lactulose breath testing may thus offer a useful measure of SIBO for identifying gut dysbiosis in PACS patients.

### 4.3. Gut microbiota and innate immunity

Type I and type III interferons (IFNs) play crucial roles in fighting viral diseases, and while type I IFN is the dominant phenotype in systemic infections, type III IFN appears to preferentially enforce and strengthen the antiviral response at mucosal sites (Mordstein et al., 2010; Pott et al., 2011). Type III IFN receptors are extensively expressed at these sites, and organs such as the gastrointestinal tract and the lungs respond strongly to systemic expression of type III IFN (Mordstein et al., 2010; Pulverer et al., 2010; Pott and Stockinger, 2017). The microbiome appears to mediate type III IFN production and induce the expression of antiviral IFN-stimulated genes in the intestinal epithelium (van Winkle et al., 2022). Importantly, mucosal microbiomes can modulate IFN responses both locally and remotely, either by systemically releasing metabolites that prime distal immune and epithelial cells, or by being sampled by immune cells that migrate to other organs and influence local immune response (Wirusanti et al., 2022). Microbial translocation itself can also drive the proliferation of altered immune IFN capabilities. For example, epithelial type III IFN promoters become suppressed and its receptors become cleaved in the presence of *Porphyromonas gingivalis* in the mouth – a strain that is capable of translocation to mucosal surfaces in the gut and respiratory tissues, where it has been associated with dysbiosis and pneumonia, respectively (Nakajima et al., 2015; Benedyk et al., 2016; Rodriguez-Hernandez et al., 2021). With growing evidence that SARS-CoV-2 increases intestinal infections, type III IFN is thus emerging as a potential therapeutic target for both acute and chronic gastrointestinal sequelae of COVID-19 (Pan et al., 2023). Importantly, type III IFN has been shown to be more potent and long-lasting than type I IFN in reducing SARS-CoV-2 viral loads in human intestinal epithelial cells (Metz-Zumaran et al., 2022).

Recently described timelines of the type I and type III IFN responses to SARS-CoV-2 infection offer insight into the mechanisms of proinflammatory cytokine storms during acute COVID-19 as well as its long-lasting effects as part of PACS. As it evolved, the SARS-CoV-2 virus developed strategies to evade and antagonize pattern recognition receptor signaling, inhibiting IFNs and preventing activation of the host innate immune response (Deng et al., 2017; Hackbart et al., 2020; Choi and Shin, 2021). Of particular interest is that this dysregulated IFN response appears to occur in the upper airways and in the early stages of COVID-19, followed by exuberant IFN production in the context of a hyperactive inflammatory response that occurs later in the lungs (Eskandarian Boroujeni et al., 2022). This reversal of the general immunity paradigm – that the IFN-mediated responses precede pro-inflammatory ones – has been shown to be specific to COVID-19, with influenza patients hospitalized for pneumonia showing traditional temporal IFN patterns (Galani et al., 2021; Kim and Shin, 2021). The delayed and exaggerated IFN activity in the acute stage of COVID-19 likely contributes its to long-term neurocognitive effects as well. Post-mortem COVID-19 analyses point to strong expression of type I IFN signatures that are specific to the choroid plexus, despite the absence of RNA and protein traces of SARS-CoV-2 in the tissue (Yang et al., 2021). Such enhanced type I IFN signaling in the choroid plexus has been reported in both healthy aging under non-infectious conditions as well as in neurodegenerative conditions such as Alzheimer’s disease – both of which are associated with decline in cognitive ability – and have been compared to PACS using other molecular signatures (Baruch et al., 2014; Stopa et al., 2018; Mavrikaki et al., 2022; Reiken et al., 2022). The choroid plexus has thus been argued as particularly susceptible to SARS-CoV-2, with the local type I IFN immune response being the possible driver of long-term cognitive deficits that are often reported in PACS (Suzzi et al., 2023). As such, this area may serve as a useful target for further inquiries into the neuropathology of PACS.

### 4.4. Intestinal barrier damage

In the context of the gut-brain PACS model, dysbiosis would presumably need to be coupled with increased gut permeability due to a compromised intestinal barrier, allowing for neuroinflammatory and neurotoxic substances such as LPS and PGN to permeate systemically. Consistent with the first prong of the proposed PACS pathogenesis along the gut-brain axis, SARS-CoV-2 has well-established effects on the intestinal epithelium that are analogous to those in the cerebrovascular endothelium. ACE2 receptors are extensively expressed by the intestinal enterocytes and serve as viral binding sites, promoting tissue-damaging interactions between SARS-CoV-2 and the epithelium (Cardinale et al., 2020; Alenazy et al., 2022). The resulting compromises to the intestinal barrier facilitate systemic permeability of potentially harmful byproducts produced by the intestinal microbiome, which in turn can influence both local and systemic inflammatory activities, further compounding the runaway inflammation of COVID-19 (Ruff et al., 2020; Delgado-Gonzalez et al., 2021). Butyrate also plays an important role in preserving the intestinal barrier. In addition to supporting of the intestinal mucosal layer, butyrate helps maintain acetylation of histones, affecting the molecular remodeling of chromatin toward a transcriptionally ready state (Candido et al., 1978). In endothelial cells specifically, butyrate effectively suppresses the expression of pro-inflammatory genes and their LPS-induced release (Chriett et al., 2019). As such, depletions of butyrate-producing bacteria have been suggested to exacerbate SARS-CoV-2-induced gut epithelial cell damage via insufficient downregulation of these mechanisms (Li et al., 2021). Butyrate has also been shown to enrich the innate immune viral response *via* the toll-like receptor signaling pathway, upregulating interleukin-1β, interferon regulatory factor-7, and interferon-alpha/beta receptor at mRNA and protein levels (Li et al., 2021). As such, butyrate appears likely to be involved in the innate immune response to SARS-CoV-2.

### 4.5. Markers of intestinal barrier damage

Intestinal fatty acid binding protein (FABP2) is expressed by intestinal enterocytes, largely in the absorptive parts of the intestinal epithelial villi, where it is involved in fatty acid absorption and transport (Gajda and Storch, 2015). While FABP2 is not a measure of transcellular permeability *proper*, such as Horseradish Peroxidase or Fluorescent Labeled Particles, it has emerged as a sensitive biomarker of intestinal epithelial dysfunction due to its physical cellular disentegration (Pelsers et al., 2003). For example, the intestinal epithelium is damaged during intestinal ischemia, and FABP2 is released into the blood, where it has been shown to reliably reflect such damage in humans (Kanda et al., 1992; Montagnana et al., 2018). Indeed, intestinal and mesenteric ischemia has recently emerged as both a presenting feature and a late complication of COVID-19 during hospitalization (Norsa et al., 2020; Kiwango et al., 2021; Singh and Kaur, 2021), with approximately one-third of such patients having ultimately succumbed to the disease (Keshavarz et al., 2021). While such severe cases warranted the computed tomography scans that ultimately revealed profound cases of gastrointestinal ischemia, increases in FABP2 have been detected in less severe COVID-19 cases as well (Prasad et al., 2021). However, there remains the question of how prevalent intestinal microinfarctions are in mild disease severities, and whether they are associated with increased FABP2 plasma levels. As such, whether the epithelial damage is sustained *via* direct viral infiltration or indirectly by intestinal ischemia, FABP2 may a highly useful metric for quantifying intestinal permeability in PACS.

Zonulin, on the other hand, is specific to paracellular epithelial damage, and is often accompanied by transcellular permeability as well, as detected by aforementioned FABP2. Zonulin modulates the abilities of tight junctions of intestinal epithelial cells to regulate paracellular permeability (Fasano et al., 2000). Specifically, increased concentrations of zonulin disassemble ‘zonula occludens’ proteins, structurally disrupting the tight junction complex (Fasano, 2012). Indeed, increased blood plasma concentrations of zonulin have been suggested to reflect intestinal permeability across several conditions (Sapone et al., 2006; Ganda Mall et al., 2018), and are closely linked to plasma concentrations of endotoxin Lipopolysaccharide and transcellular permeability biomarker FABP2 (Stevens et al., 2018). Zonulin release has additionally been implicated in gut permeability associated with celiac disease (Lammers et al., 2008) and proposed to play roles in various inflammatory and autoimmune disorders (Fasano, 2011; Asbjornsdottir et al., 2020). This leads us to speculate that similar chronic gastrointestinal disturbances in PACS patients may be due to elevated zonulin levels, with increased intestinal permeability allowing for dysbiosis-induced endotoxins to permeate systemically.

### 4.6. Systemic translocation of microbial products

Lipopolysaccharide (LPS) is a major component of the outer membrane of Gram-negative bacteria in the gut (Raetz and Whitfield, 2002), and its presence induces systemic inflammation as well as microglial activation in the brain (Dobrovolskaia and Vogel, 2002; Brown, 2019). The endotoxin has previously been found in elevated levels in hospitalized COVID-19 patients, but its presence in the post-acute disease stage and contribution to the neurocognitive PACS symptoms remains unclear (Prasad et al., 2021; Teixeira et al., 2021). Importantly, direct LPS infiltration of the brain has been demonstrated in rats, where it binds to endothelial cell receptors at blood–brain interfaces, thus further exacerbating the endothelial damage inflicted by the virus and cytokines described above (Vargas-Caraveo et al., 2017). Of particular interest is that LPS has been shown to disrupt the blood–brain barrier in some brain regions, but not in others, impacting the BBB in the thalamus, frontal cortex, cerebellum, and pons-medulla. Thalamic and frontal impairments are of particular interest in the context of PACS, as these regions are important to higher-order cognitive processes (Jung and Haier, 2007; Vakhtin et al., 2014). Systemic LPS may thus have a triple impact in post-COVID-19 pathology by stimulating pro-inflammatory cytokines and macrophage activation, directly interacting with the cerebrovascular endothelium concurrently with the virus, as well as increasing the exposure of some brain regions to neurotoxins *via* the compromised BBB.

Another common microbial byproduct that may contribute to systemic endotoxicity in PACS is peptidoglycan (PGN). While the term “endotoxin” has until recently been synonymous with LPS, PGN has emerged as a gram-positive counterpart of LPS in the recent years (Myhre et al., 2006). This was largely driven by the increasing rates of sepsis due to gram-positive organisms, which have recently overcome those due to gram-negative bacteria (Mayr et al., 2014). PGN concentrations in blood plasma have also been shown to reflect intestinal permeability due to ischemia, hemorrhagic shock, and ethanol-induced injury (Shimizu et al., 2002; Tabata et al., 2002; Tsunooka et al., 2004). Importantly, the presence of gut-derived PGN in brain dendritic cells and macrophages has been associated with idiopathic inflammatory and autoimmune conditions, such as multiple sclerosis (Schrijver et al., 2001; Visser et al., 2005). Further, the neurotoxicity of PGN has been demonstrated in rats, where it induced acute microglial and astrocytic nitric oxide production, which mediate neuronal cell death (Boje and Arora, 1992; Kim and Täuber, 1996; Buskila et al., 2005). Beyond innate immunity, PGN has also been implicated in pathogenesis of neurodevelopment and its associated disorders, such as autism spectrum disorder, which may be related to the potential cognitive sequelae of its neurotoxic properties (Arentsen et al., 2017; Gonzalez-Santana and Diaz Heijtz, 2020). Like LPS, concentrations of PGN in blood plasma have also been shown to be elevated in hospitalized COVID-19 patients (Prasad et al., 2021). As such, PGN concentration is a potential contributor to persistent neuroinflammation and neurotoxicity in the PACS framework along the gut-brain axis.

## 5. Conclusion

Here we described a comprehensive model for long COVID-19 along the gut-brain axis. In summary, neurocognitive PACS symptoms may persist because viral damage to the blood–brain and the intestinal barriers may allow for unchecked flow of harmful substances produced in the gut lumen in the context of dysbiosis. This indirect pathogenesis stands in contrast to models that ascribe the neurocognitive symptoms of PACS to direct effects of the virus, regardless of whether they occur during the acute stage or are maintained chronically by remnant viral reservoirs (Proal and VanElzakker, 2021; Swank et al., 2023). Likewise, our model also circumvents the hypothesized reactivation of existing underlying pathogens such as the Epstein–Barr virus (Peluso et al., 2023). It does, however, unify several other hypotheses for the pathogenesis of PACS and its neurocognitive symptoms, including innate immune dysregulation, impact on microbiota, as well as endothelial and epithelial damage and dysfunction that can lead to degradation of brain and intestinal barriers, respectively (Arthur et al., 2021; Haffke et al., 2022; Davis et al., 2023). Similar models along the gut-brain axis have been described previously (Vakili et al., 2022; Gareau and Barrett, 2023), and while our narrative for the initiation and maintenance of neurocognitive symptomology reflects these existing models, we also elaborate on the specific metrics that can be quantified experimentally to assess each component of the model.

The described mechanism of gut-brain pathology has been described extensively in other disorders, such as depression (Valles-Colomer et al., 2019), anxiety (Kim and Shin, 2018), neurodegenerative conditions (Ryman et al., 2023), and other chronic multi-symptom illnesses such as Gulf War illness (Alhasson et al., 2017; Bajaj et al., 2019; Keating et al., 2019). Systemic inflammation due to increased permeability in the gut has been implicated in conditions such as irritable bowel syndrome (Camilleri et al., 2012; Fukui, 2016; Moser et al., 2018), and these pathways are highly relevant to COVID-19 due to its well-established cytokine storms that can result in fatal hyper-inflammation (Cron et al., 2021). While SARS-CoV-2 infection fits particularly well into this model due to its utilization of ACE2 receptors, which are expressed extensively in the brain endothelium and the intestinal epithelium, we note that this pathogenesis may be applicable to other psychiatric as well as neurodegenerative conditions.

Multiple studies examining gut-based interventions in PACS are ongoing as of this writing, motivated by emerging data on probiotic effectiveness in alleviating COVID-19 symptoms. Blinded, randomized, and placebo-controlled probiotic treatment of symptomatic outpatient COVID-19 patients has been shown to modulate the immune response, significantly increasing SARS-CoV-2-specific IgM and IgG levels (Gutiérrez-Castrellón et al., 2022). In-patients with moderate-to-severe COVID-19 who received single-strain probiotic *Bifidobacterium* boosters were found to have significantly shorter hospital stays, IL-6 level reductions, radiological lung improvements, and lower mortality rates relative to the non-probiotic group (Bozkurt and Bilen, 2021). Probiotic fecal microbial transplantation (FMT) has been shown to produce rapid resolution of COVID-19 in acute-stage patient case reports as well (Biliński et al., 2022). Collectively, these interventions hold promise in alleviating the neurocognitive symptoms of PACS indirectly *via* the gut and in the absence of direct interventions acting on the central nervous system – as few such approaches exist currently.

## Data availability statement

The original contributions presented in the study are included in the article/supplementary material, further inquiries can be directed to the corresponding author.

## Author contributions

AP wrote the first draft of the manuscript. YM conducted literature review and organization. HL, SR, DQ, AB, and ANP contributed to the conception and design of the manuscript, and offered clinical expertise on the subject matter. AV contributed to conception and finalization of the manuscript, as well as refinement of technical and methodological sections. All authors contributed to the article and approved the submitted version.
